# The complete mitochondrial genome of *Turbo cornutus* (Trochida: Turbinidae) and its phylogeny analysis

**DOI:** 10.1080/23802359.2022.2060764

**Published:** 2022-04-08

**Authors:** Eiseul Kim, Jinhyuk Kim, Yumin Lee, Gyoungju Nah, Hae-Yeong Kim

**Affiliations:** a Institute of Life Sciences & Resources and Department of Food Science and Biotechnology, Kyung Hee University, Yongin, South Korea; b Genome Analysis Center, National Instrumentation Center for Environmental Management, Seoul National University, Seoul, South Korea

**Keywords:** *Turbo cornutus*, *Turbinidae*, complete mitochondrial genome, next-generation sequencing

## Abstract

The complete mitochondrial genome sequence of *Turbo cornutus*, a species of the *Turbinidae* family, was characterized from the *de novo* assembly of Illumina paired-end sequencing data. The complete mitochondrial genome of *T. cornutus* was 17,297 bp in length and comprised of 13 protein-coding genes, 25 tRNAs, and two rRNAs. The base composition of *T. cornutus* exhibited a high A + T content of 71.17%. The phylogenetic analysis of *T. cornutus* with 14 species from GenBank revealed that the ancestor of *Astralium haematragum* and *Bolma rugosa* was diverged from *T. cornutus*.

*Turbo cornutus* (Lightfoot, 1786) is a mollusk belonging to the family Turbinidae, distributed in relatively shallow coastal waters affected by the warm current (Kwon et al. 2010). In the case of South Korea, it is distributed mainly on Jeju Island. Previously, the horned turban was considered to be only one species of *T. cornutus* that is distributed in Korea, Japan, and China (Ozawa and Tomida 1995). However, some researchers pointed out that those from Korea and Japan, and those from China, are distinct species, and they classified the former as *T. cornutus* and identified the latter as their novel species *T. chinensis* (Ozawa and Tomida 1995; Fukuda 2017). Therefore, *T. cornutus* is restricted to southern Korea and Japan (southern part of Kyushu, Shikoku, Honshu, and Hokkaido) (Fukuda 2017). This species is a commercially important shellfish and is an edible gastropod, mainly consumed by East Asians (Kimura et al. 2010; Fukuda 2017). The complete mitochondrial genome is useful for molecular identification and understanding phylogenetic relationships (Zhang et al. 2020). Although the *T. cornutus* is an industrially important shellfish species in Korea, the complete mitogenome has not been previously elucidated. Moreover, the mitogenome of *T. cornutus* allows the prediction of genetic differences between populations in the regions along with the warm current. In this study, we sequenced the complete mitochondrial genome of *T. cornutus* collected from Korea and compared them with mitogenomes of other species.

The research materials were collected from Jeju Island, Korea (33.453611 N, 126.949167 E) in January 2021. Total genomic DNA of the specimen was extracted using DNeasy Blood and Tissue Kit (Qiagen, Hilden, Germany), according to the manufacturer’s instruction. The specimen is deposited at the National Institute of Biological Resources (NIBR, Min-Seock Do, viper@korea.kr) in Korea, under voucher number NIBRIV0000889086.

The total genomic DNA was extracted using Promega genomic wizard kit (Promega Co., Madison, WI) and the quantification and purity ratio of genomic DNA was determined using NanoDrop 2000 (Thermo Fisher Scientific, Waltham, MA). Whole genome shotgun library was constructed according to the manufacturer’s recommendation using NEXTflex^®^ Rapid DNA sequencing kit (Bioo Scientific, Austin, TX) and the library was validated using Caliper LabChip GX (PerkinElmer, Waltham, MA). The mitogenome of *T. cornutus* was sequenced by NovaSeq6000 (Illumina Inc., San Diego, CA) for 150 bp paired-end reads, generating 10.6 Gb of raw data.

After removing low-quality reads and adapters from raw data using CLC quality trim (ver. 10.0.1, CLC QIAGEN, Redwood, CA), approximately 8.9 Gb of the high quality reads were assembled by CLC Genomics Workbench (ver. 10.0.1, CLC QIAGEN, Redwood, CA) and NOVOplasty (Dierckxsens et al. 2017), followed by manual curation through PE reads mapping (Kim et al. 2015).

Annotation of the complete mitochondrial genome was performed with Mitoz (Meng et al. 2019) and manual corrections. The tRNA genes were also identified by Mitoz software. The complete mitochondrial genome sequence of *T. cornutus* was submitted to GenBank with the accession number of MZ826276.

The complete mitogenome of *T. cornutus* is 17,299 bp in length and the base composition of *T. cornutus* was estimated to be 34.98% for A, 36.18% for T, 14.94% for G, 13.88% for C with 28.83% of GC content. The mitogenome included 13 protein-coding genes (PCGs), 25 tRNA genes, two rRNA genes, and AT-rich control region. The order of gene content was described in Supplementary Table S1. The tRNA genes range from 65 to 73 bp in size. Among the mitochondrial PCGs, the ND5 is the longest, while the ATP8 is the shortest. All 13 PCGs initiate with ATG start codon and terminate with TAG (five genes) or TAA (seven genes) stop codons (Supplementary Table S1).

In order to investigate the evolutionary relationship, the complete mitochondrial genome sequences of *T. cornutus* were blasted against mitochondrial genome data in GenBank and top 13 species were retrieved from Turbinidae, Trochiae, Tegulidae, and Angariidae families. Using one outgroup species from Plakobranchidae, the complete mitochondrial genomes of 15 species including *T. cornutus* were aligned using ClustalW (ver. 2.1) (Larkin et al. 2007), followed by phylogenetic tree construction based on a maximum-likelihood (ML) analysis with Tamura-Nei Model and bootstrap value of 1000 by MEGA 7.0 (Kumar et al. 2016). The phylogenetic tree exhibited *T. cornutus* clustered with other Turbinidae species, such as *Astralium haematragum*, *Bolma rugosa*, *Lunella granulata*, *Lunella correensis*, validating *T. cornutus* is a member of Turbinidae family (Figure 1).

**Figure 1.**
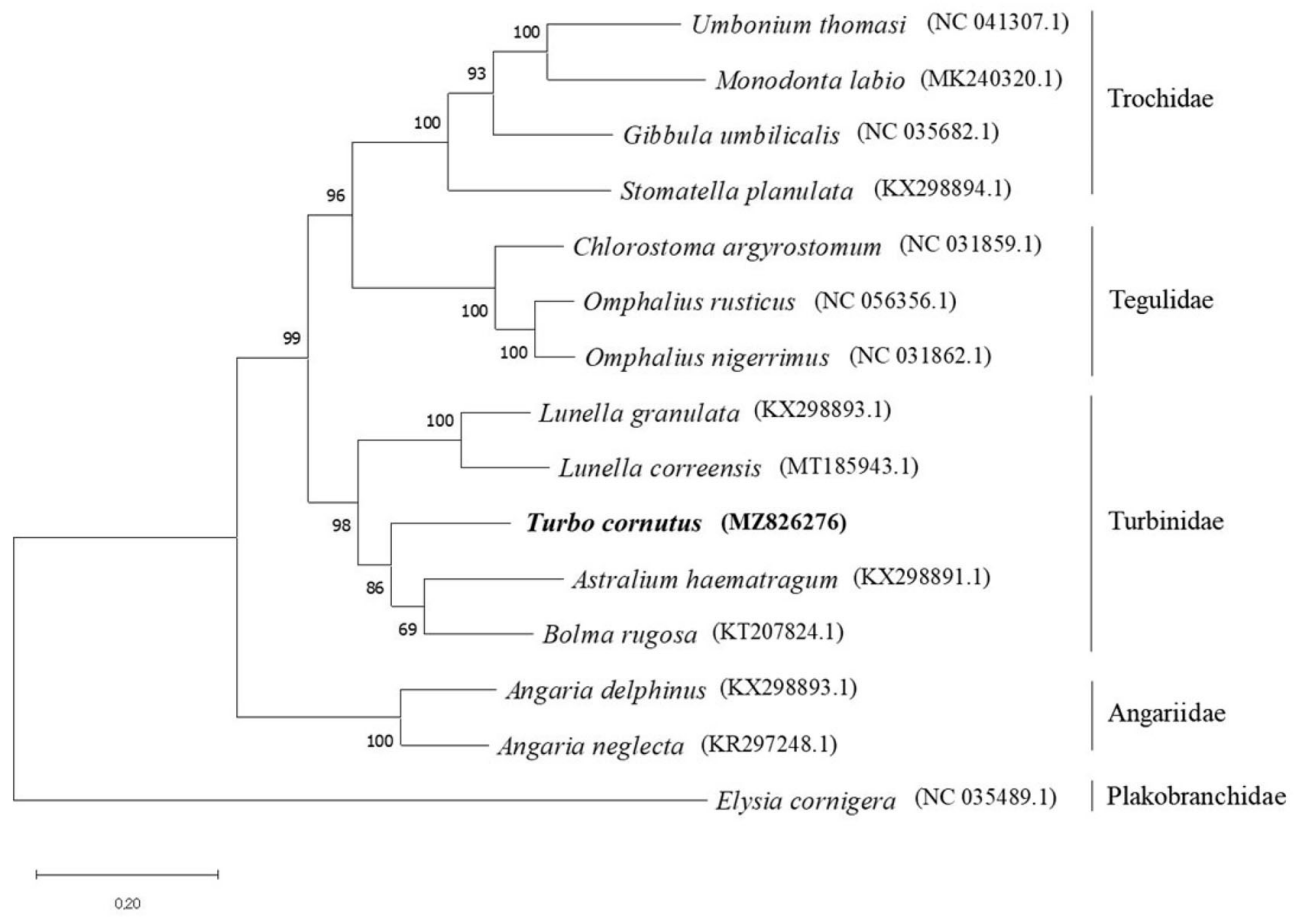
Phylogenetic relationship of *Turbo cornutus* with 14 species (including one outgroup) using complete mitochondrial genome sequences from GenBank through maximum-likelihood (ML) analysis with 1000 bootstraps.

## Supplementary Material

Supplemental Material

## Data Availability

The data that support the finding of this study are publically available in GenBank at http://www.ncbi.nlm.gov/genbank/, with reference number MZ826276. The BioProject, BioSample, and SRA numbers are PRJNA755449, SAMN20822783, and SRR15496837, respectively.
