# Supplementary material for: The complete mitochondrial genome of Turbo cornutus (Trochida: Turbinidae) and its phylogeny analysis
Source: Mitochondrial DNA B Resour. 2022 Apr 8;7(4):637–9. doi: 10.1080/23802359.2022.2060764 (PMC9004515; doi:10.1080/23802359.2022.2060764)
Supplement: Supplemental Material [file TMDN_A_2060764_SM3749.docx]

**Supplementary Table S1.** Mitochondrial genome characteristics of *Turbo cornutus*

| Gene | Position | | Size (bp) | Codon | | Intergenic nucleotides (bp) |
| --- | --- | --- | --- | --- | --- | --- |
|  | From | To |  | Start | Stop |  |
| *COX1* | 1 | 1536 | 1536 | ATG | TAA | 145 |
| *COX2* | 1682 | 2371 | 690 | ATG | TAG | 103 |
| *tRNA^Asp^* | 2475 | 2543 | 69 |  |  | 1 |
| *ATP8* | 2545 | 2724 | 180 | ATG | TAA | 127 |
| *ATP6* | 2852 | 3547 | 696 | ATG | TAA | 31 |
| *tRNA^Phe^* | 3579 | 3647 | 69 |  |  | 87 |
| *ND5* | 3735 | 5474 | 1740 | ATG | TAG | 0 |
| *tRNA^His^* | 5475 | 5540 | 66 |  |  | 64 |
| *ND4* | 5605 | 6996 | 1392 | ATG | TAA | -7 |
| *ND4L* | 6990 | 7289 | 300 | ATG | TAA | 70 |
| *tRNA^Ser(UGA)^* | 7360 | 7426 | 67 |  |  | 17 |
| *CYTB* | 7444 | 8583 | 1140 | ATG | TAA | 95 |
| *ND6* | 8679 | 9185 | 507 | ATG | TAA | 4 |
| *tRNA^Pro^* | 9190 | 9258 | 69 |  |  | 148 |
| *ND1* | 9407 | 10354 | 948 | ATG | TAA | 1 |
| *tRNA^Leu(UAA)^* | 10356 | 10423 | 68 |  |  | 37 |
| *tRNA^Leu(UAG)^* | 10461 | 10528 | 68 |  |  | 14 |
| *l-rRNA* | 10543 | 12060 | 1518 |  |  | 42 |
| *tRNA^Val^* | 12103 | 12169 | 67 |  |  | 6 |
| *s-rRNA* | 12176 | 13155 | 980 |  |  | 14 |
| *tRNA^Met^* | 13170 | 13239 | 70 |  |  | 22 |
| *tRNA^Tyr^* | 13262 | 13329 | 68 |  |  | -1 |
| *tRNA^Cys^* | 13329 | 13393 | 65 |  |  | 2 |
| *tRNA^Trp^* | 13396 | 13464 | 69 |  |  | 0 |
| *tRNA^Gln^* | 13465 | 13533 | 69 |  |  | 23 |
| *tRNA^Gly(UCC)^* | 13557 | 13612 | 56 |  |  | 3 |
| *tRNA^Glu(UUC)^* | 13616 | 13686 | 71 |  |  | 186 |
| D-loop | 13873 | 13940 |  |  |  | 156 |
| *tRNA^Glu(UUC)^* | 14097 | 14167 | 71 |  |  | 3 |
| *tRNA^Gly(UCC)^* | 14171 | 14240 | 70 |  |  | 21 |
| *COX3* | 14262 | 15041 | 780 | ATG | TAG | 35 |
| *tRNA^Lys^* | 15077 | 15138 | 62 |  |  | 0 |
| *tRNA^Ala^* | 15139 | 15206 | 68 |  |  | 17 |
| *tRNA^Arg^* | 15224 | 15292 | 69 |  |  | 44 |
| *tRNA^Asn^* | 15337 | 15408 | 72 |  |  | 20 |
| *tRNA^Thr^* | 15429 | 15501 | 73 |  |  | 43 |
| *tRNA^Ile^* | 15545 | 15612 | 68 |  |  | 5 |
| *ND3* | 15618 | 15971 | 354 | ATG | TAG | 82 |
| *tRNA^Ser(GCU)^* | 16054 | 16121 | 68 |  |  | 3 |
| *ND2* | 16125 | 17273 | 1149 | ATG | TAG |  |
